# Impact of comorbidity on health-related quality of life among type 2 diabetic patients in primary care

**DOI:** 10.1017/S1463423620000055

**Published:** 2020-04-06

**Authors:** Sandipana Pati, Sanghamitra Pati, Marjan van den Akker, F. (François) G. Schellevis, Sunita Jena, Jako S. Burgers

**Affiliations:** 1 Centre for Chronic Conditions and Injuries, Public Health Foundation of India, New Delhi, India; 2 Indian Institute of Public Health Bhubaneswar, Bhubaneswar, Odisha, India; 3 Department of Health Research, Indian Council of Medical Research Regional Medical Research Centre, Bhubaneswar, Odisha, India; 4 Institute of General Practice, Johann Wolfgang Goethe University, Frankfurt am Main, Germany; 5 Department of Family Medicine, Maastricht University, Maastricht, The Netherlands; 6 Academic Centre of General Practice, KU Leuven, Leuven, Belgium; 7 Department of General Practice and Elderly Care Medicine, Amsterdam Public Health Research Institute, Amsterdam University Medical Centers location VUmc, Amsterdam, The Netherlands; 8 NIVEL (Netherlands Institute for Health Services Research), Utrecht, The Netherlands; 9 Department of Public Health, Utkal University, Bhubaneswar, Odisha, India; 10 Department of Family Medicine, School CAPHRI, Maastricht University, Maastricht, The Netherlands; 11 Dutch College of General Practitioners, Utrecht, The Netherlands

**Keywords:** comorbidity, health-related quality of life, primary care, SF-12, type 2 diabetes

## Abstract

**Background:**

Health-related quality of life (HRQL) is an important outcome for chronic diseases such as diabetes mellitus that is associated with complications, comorbidities, and lifelong care.

**Objectives:**

The present study aims to explore the impact of comorbidities on the different dimensions of HRQL among type 2 diabetic patients attending primary care.

**Methods:**

A total of 912 type 2 diabetic patients attending primary care centers in India were assessed using a predesigned and pretested questionnaire – *Diabetes Comorbidity Evaluation Tool in Primary Care*. The HRQL was measured by physical and mental health summary scores [physical component summary (PCS) and mental component summary (MCS)] of the Short Form Health Survey 12. The associations of sociodemographic variables and clinical variables with PCS and MCS were assessed, and a minimal difference of 5 in the scores (on a scale of 0–100) was kept as clinically relevant difference for this study. Mean differences in mental (MCS) and physical (PCS) scores of quality of life by number and type of comorbid conditions in type 2 diabetic patients were calculated.

**Result:**

The presence of comorbid conditions was associated with lower scores of PCS and MCS (*P* < 0.001). Significant reduction in HRQL was found with increase in number of comorbid conditions, and negative association was established between the number of comorbidities and the PCS (*r* = −0.25, *P* < 0.0001) and MCS scores (*r* = −0.21, *P* < 0.0001). Among comorbidities, acid peptic disease, chronic lung disease, visual impairment, depression, and stroke had significantly and clinically relevant reduced scores. Duration of diabetes, use of insulin, and obesity were also associated with poor HRQL.

**Conclusion:**

Comorbidities considerably impair the HRQL among type 2 diabetic patients. National programs designed for diabetes management should also take into account the challenges of coexisting chronic conditions and its substantial effect on HRQL.

## Introduction

Health-related quality of life (HRQL) is a societal relevant measure to assess the outcomes of clinical trials and quality of care. It acts as a valuable add-on in guiding medical treatment and research, particularly in patients with chronic diseases. Studies around the globe demonstrated that chronic conditions and multimorbidity are associated with poor HRQL (Fortin *et al.*, 2006; Agborsangaya *et al.*, 2013). Among chronic diseases, diabetes mellitus is one of the most frequent chronic and debilitating diseases that demands prolonged medication use and lifestyle changes. Diabetes is associated with low HRQL in comparison to people without chronic conditions (Néss *et al.*, 1995; Rubin and Peyrot, 1999). Sprangers *et al.* (2000) concluded that quality of life among people with diabetes is comparable to people with other chronic conditions such as cardiovascular disease, cancer, visual impairment, or chronic respiratory disease. Low HRQL affects diabetes management and control, and poor glycemic control affects HRQL making it a vicious circle. The associated complications and comorbidities further affect the HRQL among diabetic patients. Adriaanse *et al.* (2016) found a high prevalence of comorbidities among type 2 diabetes mellitus (T2DM) patients in the Netherlands and its considerable impact on quality of life. Wee *et al.* (2005) concluded from a multiethnic, population-based study that coexisting chronic conditions have an additive effect on lowering of HRQL among diabetic patients. Wexler *et al.* (2006) also found that chronic complications of diabetes are associated with decreased HRQL. However, there is paucity of studies exploring the impact of multiple comorbidities on the physical and mental components of quality of life. Furthermore, the burden of diabetes is assumed to be higher in low- and middle-income countries (LMICs). Studies conducted in these countries, however, only focused on prevalence of comorbidities or on the effect of chronic complications on quality of life (Javanbakht *et al.*, 2012; Shim *et al.*, 2012; Patel *et al.*, 2014; Saleh *et al.*, 2015). The present study aims to assess the impact of the number and type of comorbid conditions on HRQL among type 2 diabetic patients attending inner-city primary care facilities in Bhubaneswar, India.

## Methods

### Study design and setting

A cross-sectional interview survey was conducted in all 17 urban primary health care centers in Bhubaneswar, the capital city of Odisha (India) with a population of 900 000 inhabitants (Anon, 2015). According to the National Sample Survey Office’s 71st round on social consumption of health, about 72% of outpatient care in Odisha is provided by public health care professionals (Sundararaman *et al.*, 2016). The public health care system has a three-tier structure comprising of primary, secondary, and tertiary levels. Primary health care centers are involved in delivering primary care, while district hospitals and sub-divisional hospitals render secondary care. Tertiary health care is provided by medical college hospitals.

### Study participants

Patients attending a primary health care center between September 2014 and February 2015, who had been diagnosed by a physician with T2DM for more than 6 months according to their personal medical record, were eligible to be included in the study. Minimum sample size required was estimated to be 942. This was based on prior studies (Struijs *et al.*, 2006) of an expected prevalence of 40% [confidence interval (CI) = 95%], an *α* value of 0.05 and power at 0.8, with confidence levels set at 95%, and a non-response rate of 10%.

#### Sampling

A multistage sampling design was used. All the public primary health care facilities in the city of Bhubaneswar were included in the study. The calculated required sample size was divided between these 17 centers based on the proportional allocation method, weighted depending on the average outpatient attendance of the past 6 months. Owing to limited consultation time in the health care centers, and only one interviewer being available per center, and each interview duration being 20–30 min, for the feasibility of the study every third eligible T2DM patient was invited. Patients too ill to participate or with emergency health conditions were excluded from the study. Anonymized details of all patients who refused to participate (age, gender, reason for exclusion) were recorded to compare the characteristics of the participants with the nonparticipants.

### Measurements

Participating patients were interviewed in a separate private chamber using a predesigned and pretested questionnaire **–** *Diabetes Comorbidity Evaluation Tool in Primary Care (DCET-PC)*. The DCET-PC is derived from the ‘Multimorbidity Assessment Questionnaire for Primary Care’, a validated questionnaire, which was pretested and adapted for our study according to the feedback from pretesting (Pati *et al.*, 2016). Two graduate nurses trained in patient history taking and interview techniques carried out the interviews, and 10% of the interviews were carried out in the presence of the first author. The DCET-PC (Supplementary Material) included questions on sociodemographic variables, that is, age, sex, socioeconomic status (above poverty line, below poverty line), educational level (no education, primary level, secondary, graduate, and above), employment status (employed, unemployed, homemaker, retired), and clinical variables (Kung *et al.*, 1996; Janus *et al.*, 2000; Shim *et al.*, 2012) such as the existence of comorbid conditions, eliciting information on whether the patient had any of the 15 listed chronic conditions other than diabetes and obesity [defined as body mass index (BMI) >25, collected by anthropometry measurement], duration of diabetes (IASO, 2000), and type of diabetes treatment (oral medication, insulin use). The details of development and domains of the DCET-PC questionnaire are described in our previous paper (Pati and Schellevis, 2017).

#### Health-related quality of life

HRQL was measured using the Short Form Health Survey 12 (SF-12), which is a shorter version of the 36-item SF-36 Health Survey. This includes the measurement of physical functioning, role physical, role emotional, bodily pain, general health, vitality, social functioning, and mental health (MH). The eight domain scores were combined into the SF-12 physical component summary (PCS-12) and mental component summary (MCS-12) scores. The summary scores of PCS and MCS, which were derived by the weighted sum of 12-item scores using the US standard SF-12 scoring algorithm, were considered as the principal outcomes of this study (Ware *et al.*, 1995; Lam *et al.*, 2005).

### Statistical analysis

Characteristics of the study samples are described using means and SDs, or proportions and 95% CIs, where appropriate. Descriptive statistics were performed with HRQL and clinical characteristics. Differences in study sample characteristics for patients with and without comorbidities were examined using *t*-tests for continuous variables and chi-square tests for dichotomous and categorical variables. The associations of sociodemographic variables and clinical variables with PCS and MCS were assessed, and a minimal score difference of 5 (on a scale of 0–100) was kept as a clinically relevant difference for this study. Pearson’s correlations test was performed in order to assess the linear relationship and strength of association between the number of comorbidities and quality of life. To analyze both parameters, we adjusted for variables such as age, sex, duration of diabetes, BMI, and insulin use and calculated the unadjusted and adjusted *β* coefficients for MCS and PCS scores by number of comorbid conditions [0 (reference category), 1, 2, 3, and 4 or more] among T2DM patients. Additionally, we performed analysis of variance to test the differences in unadjusted and adjusted mean PCS and MCS scores by number of comorbid chronic conditions. Furthermore, mean differences in mental (MCS) and physical (PCS) scores of quality of life by comorbid chronic condition in type 2 diabetic patients were calculated. Statistical analyses were performed using STATA V12 (Stata Crop V12, Texas, USA).

### Ethical considerations

Respondents were informed about the purpose of the study and the information assessed. We collected their signature or thumb impression on the informed consent form. The data were coded and the identities of the respondents were kept confidential. The Odisha state research and ethics committee gave the ethical approval for the study (letter no. 161/SHRMU dt. 16.05.2014).

## Results

A total of 942 T2DM patients were approached with a response rate of 97% (*n* = 912). The age group of 50–59 years (34.5%) was the largest, and the mean age of respondents was 55.3 (SD: 10.3) years. Most participants were male (63.1%), had university education (40.2%), and were living below poverty line (63.3%). The overall PCS (mean ± SD) was 32.8 ± 13.7 and the overall MCS (mean ± SD) was 45.9 ± 9.0. The characteristics of study participants are summarized in Table 1.

**Table 1.** Basic characteristics of type 2 diabetic patients by comorbidity status

	Total (%) (*n* = 912)	Without comorbidity (*n* = 146) % [95% CI]	With comorbidity (*n* = 766) % [95% CI]
Age group (years)			
18–29	3 (0.3)	1.3 [0.01–3.2]	0.1 [0.01–0.4]
30–39	61 (6.7)	8.7 [4.1–13.2]	6.3 [4.5–8.0]
40–49	198 (21.7)	28.7 [21.4–35.9]	20.3 [17.4–23.1]
50–59	314 (34.5)	39.3 [31.5–47.2]	33.4 [30.1–36.7]
60–69	249 (27.3)	17.3 [11.2–23.4]	29.5 [26.2–32.7]
≥70	87 (9.5)	4.7 [1.3–8.1]	10.5 [8.3–12.6]
Gender			
Male	575 (63.1)	74.0 [66.9–81.0]	61.0 [57.5–64.5]
Female	337 (36.9)	26.0 [18.9–33.0]	39.0 [35.5–42.5]
Socioeconomic status			
Above poverty line	334 (36.6)	36.2 [24.8–47.7]	70.5 [66.4–74.6]
Below poverty line	578 (63.4)	63.8 [52.3–75.2]	29.5 [25.4–33.6]
Highest education			
Illiterate	75 (8.2)	8.7 [4.1–13.2]	8.4 [6.4–10.3]
Primary	155 (17.0)	22.0 [15.3–28.7]	16.0 [13.4–18.6]
Secondary	315 (34.5)	32.7 [25.1–40.2]	34.8 [31.4–38.2]
University	367 (40.3)	36.7 [28.9–44.4]	40.8 [37.3–38.2]
Risk factor: body mass index			
Underweight	80 (8.8)	4.7 [1.3–8.1]	2.1 [1.1–3.1]
Normal	251 (27.6)	40.0 [32.1–47.9]	20.0 [17.2–22.9]
Overweight	166 (18.2)	19.3 [13.0–25.7]	19.4 [16.5–22.2]
Obese	415 (45.4)	36.0 [28.3–43.7]	58.5 [55.0–62.0]
Oral diabetic medication			
Yes	751 (82.4)	82.8 [75.7–88.6]	82.2 [79.3–84.8]
No	161 (17.6)	17.1 [11.3–24.2]	17.7 [15.5–20.6]
Insulin use			
Yes	105 (11.5)	16.4 [10.8–23.4]	10.6 [8.4–12.9]
No	807 (88.5)	83.6[76.5–89.2]	89.4[87.0–91.5]
Occupation			
Unemployed	80 (8.8)	3.4[1.1–7.8]	9.8[7.7–12.1]
Homemaker	252 (27.6)	21.2[14.9–28.7]	28.8[25.6–32.2]
Retired	166 (18.2)	17.8[11.9–24.9]	18.3[15.6–21.1]
Employed	414 (45.4)	57.5[49–65.6]	43.1[39.5–46.6]
Duration of disease			
≤5 years	454 (49.8)	54.1[45.6–62.3]	48.9[45.3–52.5]
>5 Years	458 (50.2)	45.8[37.6–54.3]	51.1[47.4–54.6]
SF-12 Health Survey			
PCS-12 (mean ± SD)	32.8 ± 13.6	37.2 ± 14.3	32.0 ± 13.4
MCS-12 (mean ± SD)	45.9 ± 9.0	49.5 ± 10.3	45.2 ± 8.7

CI = confidence interval; SF-12 = F-Short Form Health Survey 12; PCS-12 = physical component summary 12; MCS-12 = mental component summary 12.

### Prevalence of comorbidities

**Figure 1.**
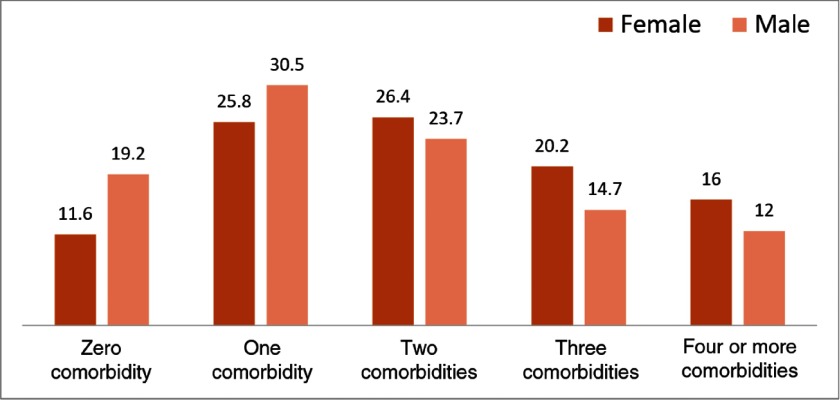
Number of comorbidities across sex.

**Figure 2.**
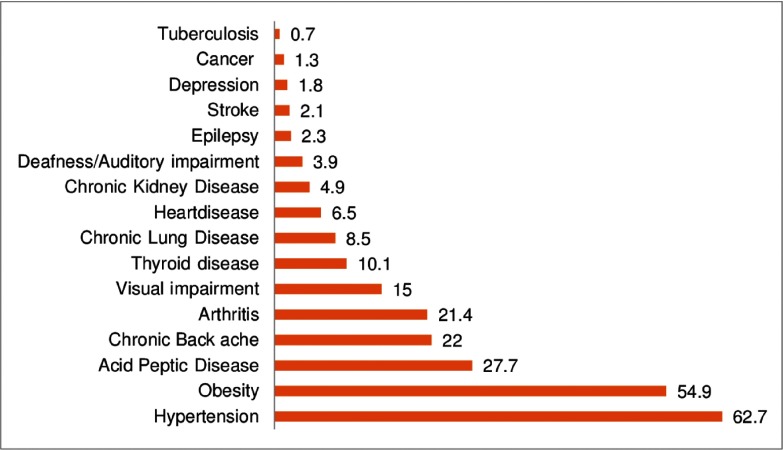
Chronic comorbidities.

The mean number of comorbidities was 1.8 (SD: 0.4). Among the participants, 84% had any comorbidity, and the majority of participants had a single (29%) comorbidity, followed by 25% with two, 17% with three, and 14% with four or more comorbidities. Female patients reported more comorbidities than male patients (Figure 1). The most frequent comorbid conditions among participants were hypertension (62%), followed by acid peptic disease (APD; 27%), chronic back ache (21%), and arthritis (21%) (Figure 2).

### Quality of life related to number of comorbid conditions

As the number of comorbidities increased, the mean difference in PCS and MCS unadjusted and adjusted scores significantly increased, indicating lower physical and mental HRQL. These effects were most pronounced for the PCS. The unadjusted and adjusted PCS mean score differences increased to −11.2 and −9.8, respectively, for diabetic patients with four or more comorbid conditions compared to patients without comorbid conditions (Table 2). The mean score decreased in both PCS and MCS as the number of comorbid conditions increased. Additional Pearson’s correlations confirmed the negative association between the number of comorbidities and the PCS (*r* = −0.25, *P* < 0.0001) and MCS scores (*r* = −0.21, *P* < 0.0001)

**Table 2.** Mean scores with SD, unadjusted and adjusted score differences (95% confidence interval) for PCS and mental MCS scores by number of comorbid conditions among type 2 diabetic patients (*n* = 912)

Number of comorbidities	PCS			MCS		
	Mean score ± SD	Mean difference (unadjusted)	Mean difference (adjusted)^a^	Mean score ± SD	Mean difference (unadjusted)	Mean difference (adjusted)^a^
0 (*n* = 146)	37.2 ± 14.3	Ref.	Ref.	49.5 ± 10.3	Ref.	Ref.
1 (*n* = 265)	34.9 ± 14.7	−2.3 (−4.9 to 0.4)	−2.8 (−5.3 to −0.3)	46.4 ± 9.6	−3.1 (−5.0 to −1.4)	−3.8 (−5.5 to −2.0)
2 (*n* = 226)	32.8 ± 12.9	−4.4 (−7.1 to −1.6)	−4.1 (−6.8 to −1.5)	45.7 ± 8.5	−3.8 (−5.6 to −2.0)	−4.1 (5.9 to −2.3)
3 (*n* = 152)	29.8 ± 11.2	−7.4 (−10.3 to −4.4)	−6.7 (−9.6 to −3.7)	44.5 ± 7.3	−5.0 (−7.0 to −3.0)	−5.3 (−7.3 to −3.3)
4 or more (*n* = 123)	26.1 ± 10.7	−11.2 (−10.4 to −8.0)	−9.8 (−13.0 to −6.6)	42.8 ± 7.6	−6.7 (−8.9 to −4.6)	−7.2 (−9.4 to −4.9)

PCS = physical component summary; MCS = mental component summary.

a Adjusted for age, sex, duration of diabetes, body mass index, and insulin use.

### Quality of life related to clinical characteristics

The PCS and MCS scores with respect to clinical characteristics are presented in Table 3. The presence of comorbid conditions was associated with lower scores of PCS and MCS (*P* < 0.001). The mean duration of diabetes for respondents was 7.1 (SD: 5.8) years. The PCS was lower in patients with a duration of disease >5 years (31.2 ± 14) than in patients having a duration of disease <5 years (34.4 ± 13.1). In contrast, no statistically significant difference was found on the MCS related to duration of disease. T2DM patients taking insulin injection had a significantly lower MCS score (44.0 ± 10) than those not taking insulin injection (48.1 ± 8.9). The mean BMI was 26.0 kg/m^2^ (SD: 4.9), and the higher the BMI the poorer the score of both PCS and MCS.

**Table 3.** Descriptive statistics of health-related quality of life scores, PCS-12, and MCS-12 by clinical characteristics

	Total = 912	PCS-12		MCS-12	
		Mean ± SD	*P*-value	Mean ± SD	*P*-value
Comorbidities					
Absent (%)	146 (16.4)	37.2 ± 14.3	<0.001	49.5 ± 10.3	<0.001
Present (%)	766 (83.5)	32.0 ± 13.4		45.2 ± 8.7	
Duration of type 2 diabetes mellitus					
Duration 5 years (%)	454 (49.8)	34.4 ± 13.2	<0.001	46.2 ± 8.6	0.484
Duration >5 years (%)	458 (50.2)	31.2 ± 14		45.7 ± 9.5	
Drug treatment					
No insulin injection (%)	807 (88.5)	31.4 ± 13.4	0.026	44.0 ± 10	0.003
Insulin injections (%)	105 (11.5)	28.4 ± 14.5		48.2 ± 8.9	
Body mass index (kg/m^2^)					
Underweight (%)	29 (2.5)	27.4 ± 15.8	<0.001	43.2 ± 11.5	<0.001
Normal	211 (23.1)	28.8 ± 14.4		43.6 ± 10	
Overweight	177 (19.4)	32.0 ± 13.7		44.8 ± 9.0	
Obese	501 (54.9)	35 ± 12.7		47.4 ± 8.2	

PCS-12 = physical component summary; MCS-12 = mental component summary.

### Quality of life related to type of comorbidity

Most comorbid conditions were associated with decreased physical HRQL, with the exception of deafness/auditory impairment, epilepsy, depression, and cancer. Stroke had highest negative impact on physical HRQL with a mean difference of 11.9, followed by visual impairment (5.6), chronic lung disease (5.9), chronic kidney disease (5.5), heart disease (5.5), and APD (5.4). Most comorbid conditions were also associated with decreased mental HRQL but the differences were small. Only depression showed a clinically relevant negative impact on MCS, with a mean difference score of 6.8. (Table 4)

**Table 4.** Mean differences in PCS and MCS scores between type 2 diabetic patients with and without the comorbid condition

Comorbid condition	*n*	Mean difference*	
		PCS	MCS
Hypertension	572	1.3*	1.0*
Obesity	501	4.8*	3.3*
Acid peptic disease	253	**5.4** *	3.9*
Chronic back ache	200	3.3*	2.8*
Arthritis	196	4.8*	2.4*
Visual impairment	137	**5.6** *	1.9
Thyroid disease	92	0.3*	−0.3*
Chronic lung disease	78	**5.9** *	2.8*
Heart disease	60	**5.5** *	2.8*
Chronic kidney disease	45	**5.5** *	2.4*
Deafness/auditory impairment	36	**8.2**	4.8
Epilepsy	21	−1.6	−0.1
Stroke	20	**11.9** *	**5.1**
Depression	17	−0.5	**6.8** *
Cancer	12	4.3	0.3
Tuberculosis	7	**−7.8** *	−3.2

PCS = physical component summary; MCS = mental component summary.

Clinically relevant mean differences, defined as ≥5, are presented in bold.

* Statistically significant with *P* < 0.05.

## Discussion

Diabetes mellitus is known to be associated with reduced HRQL due to its chronicity and complications. In this context, the findings of the present study are important as it explores the impact of various patient characteristics and comorbid conditions on both physical and mental components of HRQL among type 2 diabetic patients attending primary care. This study found that the presence of comorbidity affected the PCS and MCS significantly among diabetic patients. Previous studies have also concluded that quality of life among diabetic patients with comorbid conditions is lower than individuals with only diabetes (Maddigan *et al.*, 2005).

An important finding is the significant association of the comorbidity count with the HRQL. With the increase in number of comorbid conditions, there was a considerable reduction of both components of HRQL showing that a dose–response relationship is likely. Like past studies, our study has also reinforced that there is greater deterioration of the physical component compared to the mental component with the rise in number of comorbidities (Jasani *et al.*, 1999; Fortin *et al.*, 2006; Wang *et al.*, 2008).

Among comorbid conditions studied, hypertension was the most prevalent condition. The differences of PCS and MCS scores with patients without hypertension were low and statistically significant but were not clinically relevant. A possible explanation could be the asymptomatic nature of hypertension. Similar findings have also been reported by other studies (Stewart *et al.*, 1989; Wood *et al.*, 1998).

In our study, we found a substantial impact of stroke on both PCS and MCS. Stroke or cerebrovascular attack is for the most part of a debilitating disease. Multiple studies also concluded the reduced quality of life associated with stroke (Sprangers *et al.*, 2000; Oliva *et al.*, 2012).

APD was significantly associated with lower PCS and MCS, similar to prior studies. Earlier studies have stated that aggravation of symptoms such as heartburn with simple physical activities like bending forward or moderate housework negatively influences the physical component scores (Jasani *et al.*, 1999; Alonso *et al.*, 2004).

Visual and auditory impairment had a high impact on HRQL due to limitation to mobility and daily activities. Our study confirmed that visual impairment affects the physical component of HRQL. The impact of auditory impairment and deafness on physical component of HRQL was not statistically significant in our study, which may be due to the low number of diabetic patients with this comorbidity limiting the power of the study. Previous studies also concluded that sensory impairment has a negative influence on activities of daily living and considerably affects HRQL (Langelaan *et al.*, 2007).

Musculoskeletal disorders like chronic back ache and arthritis were associated with poor HRQL. However, contrary to previous studies, the mean score differences in our study were not clinically relevant. A significant and clinically relevant association was found between chronic lung disorders and PCS, similar to the conclusions from previous studies (Ketelaars *et al.*, 1996; Eisner *et al.*, 2008 ; Stridsman *et al.*, 2015). The reduced HRQL scores in patients with chronic kidney disease and heart disease are also consistent with findings from previous studies (Yong *et al.*, 2009; Soni *et al.*, 2010; y Pena *et al.*, 2010)

Obesity was not considered as comorbidity in this study as it is strongly related to diabetes mellitus. As in past studies, HRQL among diabetic patients with obesity was lower than in diabetic patients with normal weight (Wong *et al.*, 2013), but the difference was not found to be clinically relevant in our study. A possible explanation could be the coexistence of other comorbidities with higher impact on HRQL.

Consistent with prior findings (Zurita-Cruz *et al.*, 2018), diabetic patients with depression had poor MCS scores, but depression did not affect the PCS. Other mental conditions than depression were not included in our study.

### Strength and limitations

This is the first study to assess the impact of comorbidity on HRQL among type 2 diabetic patients in urban primary care settings in India. As it is a primary care facility study, the findings can be fairly generalized. The robust sample size of our study is another strength. A wide range of comorbidities, including concordant and discordant comorbidities, have been taken into account for studying the impact. The findings of our study can act as a base for future longitudinal studies to explore in greater detail the impact of comorbid conditions on HRQL among T2DM patients.

As a cross-sectional study, the lack of causality and effect explanation is its major limitation. The issues of reliability and validity with self-reported comorbidity are another limitation of our study. HRQL is a subjective issue, and as this is a point-in-time study, it is possible that if the respondents were interviewed at other times, there may be variation in responses. Lack of data on the severity of comorbid conditions is also a limitation as the severity of a condition plays an important role in the HRQL. Age and duration of disease may be considered as proxy measures for severity for which we did adjusted analyses.

### Policy implications

The present study calls attention to the adverse impact of comorbid conditions on HRQL among diabetic patients. It reiterates the need for focused and comprehensive care for chronic conditions especially diabetes mellitus, which is associated with multiple complications and comorbidities. As accessible and affordable first level of care, primary health centers (PHCs) are ideally placed for management of multiple chronic conditions. However, in LMICs like India where most of the PHCs are ill-equipped, the chronic disease care is generally fragmented and adds to the burden of chronic disease patients. The recent ‘Health and Wellness Centre’ (HWC) initiative of Government of India, wherein existing PHCs shall be strengthened with facilities for comprehensive primary care to improve community access, is a step forward in the direction of continuity of care (NHSRC, 2018). However, other than screening and prevention programs, that is, existing National Programme for the Prevention and Control of Cancer, Diabetes, Cardiovascular Diseases and Stroke, due emphasis should also be placed on the curative and rehabilitative aspects of the chronic conditions. As concluded in our study, diabetic patients with visual impairment and stroke have significantly reduced quality of life, hence more emphasis may be given to the rehabilitation of the sequelae of these diseases to reduce the burden and improve quality of life and health outcomes. The provision of physiotherapist at PHCs under HWC initiative is a welcome step in this regard. MH counselors can play a significant role in addressing depression related to chronic conditions among these patients. Hence, we recommend the integration of National Mental Health Programme components for primary care with the HWC initiative that will help in improving the quality of life and health outcomes among diabetic patients.

## Supplementary material

For supplementary material accompanying this paper visit http://dx.doi.org/10.1017/S1463423620000055. click here to view supplementary material
